# The Involvement of the *csy1* Gene in the Antimicrobial Resistance of *Acinetobacter baumannii*

**DOI:** 10.3389/fmed.2022.797104

**Published:** 2022-01-26

**Authors:** Tingting Guo, Xiaoli Sun, Mengying Li, Yuhang Wang, Hongmei Jiao, Guocai Li

**Affiliations:** ^1^Department of Microbiology, School of Medicine, Yangzhou University, Yangzhou, China; ^2^Jiangsu Key Laboratory of Zoonosis/Jiangsu Co-Innovation Center for Prevention and Control of Important Animal Infectious Diseases and Zoonoses, Yangzhou University, Yangzhou, China; ^3^Jiangsu Key Laboratory of Integrated Traditional Chinese and Western Medicine for Prevention and Treatment of Senile Diseases, Yangzhou, China; ^4^Department of Pharmacy, Suzhou Hospital of Integrated Traditional Chinese and Western Medicine, Suzhou, China

**Keywords:** *Acinetobacter baumannii*, nosocomial pathogen, CRISPR-Cas system, antimicrobial resistance, *csy1* gene

## Abstract

*Acinetobacter baumannii* is an important, opportunistic nosocomial pathogen that causes a variety of nosocomial infections, and whose drug resistance rate has increased in recent years. The CRISPR-Cas system exists in several bacteria, providing adaptive immunity to foreign nucleic acid invasion. This study explores whether CRISPR-Cas is related to drug resistance. Antibiotics were used to treat strains ATCC19606 and AB43, and the expression of CRISPR-related genes was found to be changed. The Csy proteins (Csy1–4) were previously detected to promote target recognition; however, the potential function of *csy1* gene is still unknown. Thus, the Rec_Ab_ homologous recombination system was utilized to knock out the *csy1* gene from *A. baumannii* AB43, which carries the Type I-Fb CRISPR-Cas system, and to observe the drug resistance changes in wild-type and *csy1*-deleted strains. The AB43Δ*csy1* mutant strain was found to become resistant to antibiotics, while the wild-type strain was sensitive to antibiotics. Moreover, transcriptome analysis revealed that the *csy1* gene regulates genes encoding CRISPR-Cas-related proteins, drug-resistant efflux pumps, membrane proteins, and oxidative phosphorylation-related proteins, inhibiting antimicrobial resistance in *A. baumannii*. The *in vitro* resistance development assay revealed that the complete CRISPR-Cas system could inhibit the development of bacterial resistance. Our findings expand our understanding of the role of CRISPR-Cas *csy1* gene in *A. baumannii* and link the CRISPR-Cas system to the biogenesis of bacterial drug-resistant structures.

## Introduction

*Acinetobacter baumannii*, a non-fermented Gram-negative bacterium, is one of the primary causes of nosocomial infections worldwide. It mainly causes ventilator-associated pneumonia and blood, urinary tract, skin, and soft tissue infections, particularly in critically ill patients in the intensive care unit (1). Over the past few years, the drug resistance rate of *A. baumannii* has gradually increased (2). Due to the treatment challenges posed by emerging and increasing drug resistance, multi-drug resistant (MDR) *A. baumannii* poses a global threat to human health (3).

The CRISPR-Cas system is an immune system used in prokaryotes resisting invasion of foreign genetic elements. It generally consists of three parts: a CRISPR array, a leader sequence, and Cas-related proteins (4). In general, three different stages have been described in the CRISPR-Cas immune response: (i) adaptation, (ii) CRISPR (Cr) RNA expression and maturation, and (iii) interference (5). The CRISPR-Cas system directs sequence-specific cleavage of phage and plasmid nucleic acids using nucleases programmed by small RNAs (6). The CRISPR-Cas system also inhibits conjugation and transformation, thereby limiting horizontal gene transfer. As the latter significantly affects bacterial evolution, the spread of antibiotic resistance and virulence determinants is the most pronounced (7). Several studies have demonstrated that the CRISPR-Cas system is associated with bacterial drug resistance. A previous study has demonstrated that a variety of genes are regulated by the *Campylobacter jejuni* II CRISPR-Cas9 system to promote bacterial drug resistance (8). The CRISPR/Cas9 system can mediate MDR *Escherichia coli* to restore antibiotic sensitivity (9). The Cas9-dependent CRISPR-Cas system of the intracellular bacterial pathogen *Francisella novicida* enhances antibiotic resistance by strengthening envelope integrity (10). In the I-F CRISPR-Cas system, multiple Cas proteins (Csy1–4) and CRISPR RNA (CrRNA) form a surveillance complex (Csy complex) for target recognition (11). Studies have shown that Csy proteins (Csy1–4) promote target recognition by enhancing sequence-specific hybridization between CRISPR RNA and complementary target sequences (12).

**Figure 1:**
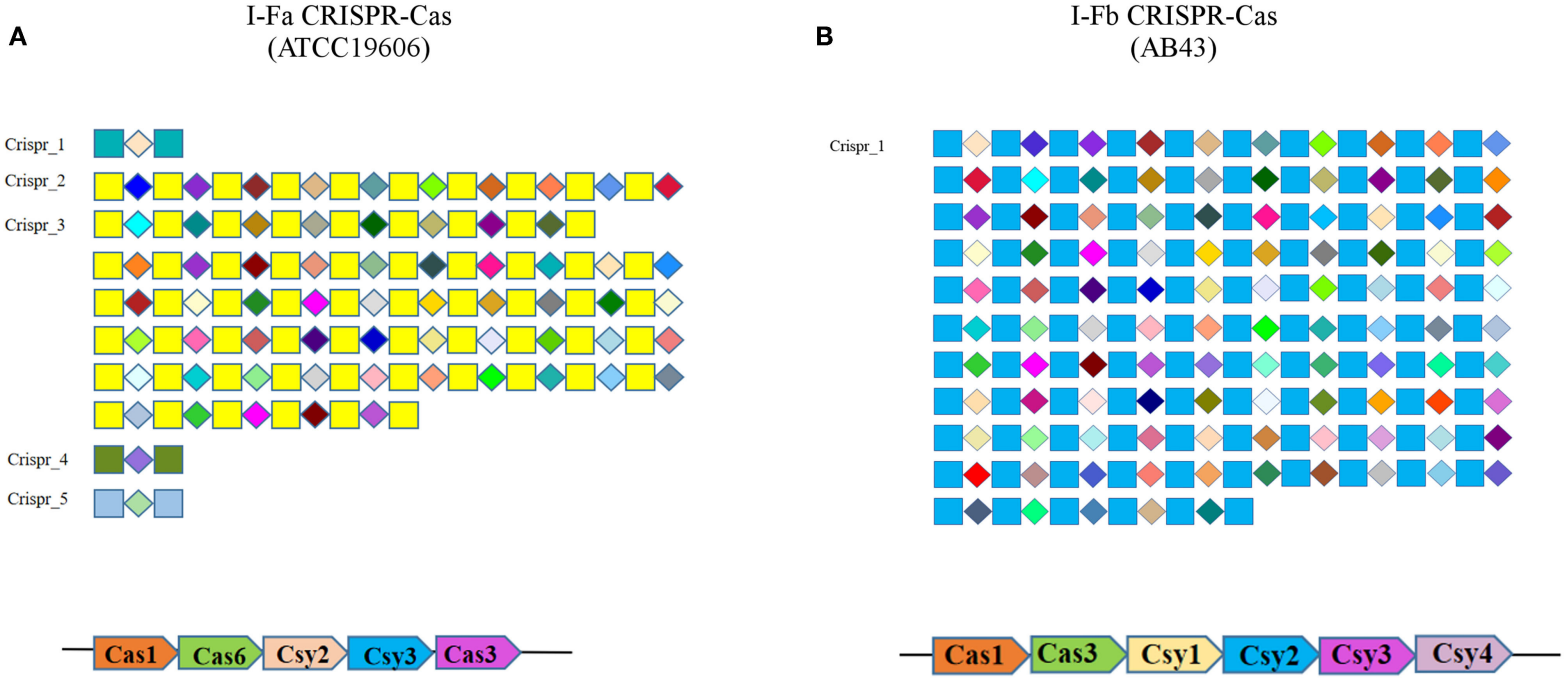
Repeat (squares), spacer (diamonds), and the composition of different types of CRISPR-cas system of A. baumannii. **(A)** ATCC19606; **(B)** AB43.

The Type I CRISPR-Cas system is the most widely distributed in nature (13). The unique feature of Type I-F CRISPR-Cas is the fusion of Cas2 and Cas3 (Cas2/3), together with Cas1 mediating the integration of the spacer into the CRISPR site (14, 15). There are two primary subtypes of the I-F CRISPR-Cas system known in *A. baumannii*, namely, Type I-Fa and Type I-Fb (16–18). The composition of Type I-Fa and Type I-Fb is illustrated in Figure 1. The Csy1 protein is missing in the Type I-Fa CRISPR-Cas system; however, the other components contain additional domains that can compensate for the role of Csy1 protein (19). Moreover, the role of *csy1* gene in antimicrobial resistance is still unknown.

Our study aimed to research whether antibiotics affect the expression of CRISPR-Cas-related genes in *A. baumannii* to determine the relationship of *csy1* gene with antimicrobial resistance.

## Materials and Methods

### Bacterial Strains and Plasmids

This study included standard strain *A. baumannii* ATCC19606 (GenBank: CP045108.1, https://www.ncbi.nlm.nih.gov/nuccore/CP045108.1), which carries the complete CRISPR-Cas subtype I-Fa system, and clinical isolates of *A. baumannii* AB43 (GenBank: CP083182.1, https://www.ncbi.nlm.nih.gov/nuccore/2095284784), carrying the complete CRISPR-Cas subtype I-Fb system. Moreover, AB133, ABF7, and ABE5 are clinical isolates containing an incomplete I-Fa system, an incomplete I-Fb system, or no CRISPR-Cas system, respectively (Table 1). The clinical isolates used in this study were isolated from the Affiliated Hospital of Yangzhou University and identified in our laboratory. The bacterial strains and plasmids used in this study are listed in Table 1.

**Table 1:** Strains and plasmids used in this study.

**Strain/plasmid**	**Relevant genotype and property**	**Source and/or reference**
*A. baumannii* strain19606	I-Fa CRISPR-Cas	ATCC
*A. baumannii* strain AB43	I-F b CRISPR-Cas	This study
*A. baumannii* strain AB133	I-F a CRISPR-Cas	This study
*A. baumannii* strain ABF7	I-F b CRISPR-Cas	This study
*A. baumannii* strain ABE5	Lack CRISPR-Cas	This study
*A. baumannii* strain AB43*Δcsy1*	Δ*csy1*	This study
*A. baumannii* strain AB43*Δcsy1/pcsy1*	Δ*csy1/*p*csy1*	This study
pMMB67EH	AMP^r^	This study
pAT04	pMMB67EH with RecAb system, Tet^r^	This study
pAT03	pMMB67EH with FLP recombinase kan^r^	This study
pKD4	Kan^r^	This study

*r, represents resistance*.

CRISPR-Cas Finder (https://crisprcas.i2bc.paris-saclay.fr/CrisprCasFinder/Index) was used to determine the existence of the CRISPR-Cas system and spacers in the genomes of strains ATCC19606 and AB43. The existence of clinical strains (AB133, ABF7, and ABE5) with CRISPR-Cas genes was determined by PCR (primers are shown in Supplementary Table 1).

### Antimicrobial Susceptibility Assessment

Strains with different CRISPR-Cas systems were subjected to determine the minimum inhibitory concentrations (MICs) of doxycycline, minocycline, tigecycline, ceftriaxone, imipenem, gentamicin, kanamycin, ciprofloxacin, polymyxin B, colistin, erythromycin, and rifampin to detect the relationship of CRISPR-Cas system integrity with drug resistance. The MICs of antibiotics were measured using the standard broth microdilution method, according to the Clinical and Laboratory Standard Institute 2020 guideline.

### Real-Time Quantitative Reverse Transcription-PCR (qRT-PCR) to Detect CRISPR Gene Expression After Exposure to Antimicrobials

Strains ATCC19606 and AB43 were grown to the early-exponential phase and incubated with the MICs of antibiotics for 4 h. Total RNA was extracted from the bacteria using a total RNA extraction kit (TIANGEN, Beijing, PR China). The HISCRIPT 1st strand cDNA synthesis kit (Vazyme, Nanjing, China) was used to synthesize cDNA. A housekeeping gene (16S rRNA gene) was used as an internal control for each sample. qRT-PCR was performed on an ABI 7,500 RT-PCR system (Applied Biosystems, CA, USA), and SYBR Green was the dye (Vazyme, Nanjing, China) utilized. Primers used for qRT-PCR are illustrated in Supplementary Table 2. The relative gene expression levels were compared with those of 16S rRNA by the 2^−ΔΔ*Ct*^ method.

### Construction of the *csy1* Gene Deletion Mutant

To delete the c*sy1* gene from *A. baumannii* AB43, pKD4 was used as a template to amplify the complete kanamycin cassette gene fragments. The PCR amplification products were identified by 1% agarose gel electrophoresis and purified using the FastPure Gel DNA Extraction Mini Kit (Vazyme). *A. baumannii* carrying RecAb on pMMB67EH (pAT04) was inoculated into liquid Luria Bertani (LB) medium containing carbenicillin to preserve the plasmid. IPTG was added to bacteria to the mid-log phase with a final concentration of 2 mM, incubated at 37°C for 3 h. Then bacteria were collected, washed thrice with 10% ice-cold glycerol, 1,000-fold concentrated. One microgram recombinant engineering PCR products were electrotransformed into *A. baumannii* AB43 competent cells (100 μL, ~10^10^ bacteria) in 2 mm cuvette at 1.8 kV. The bacteria are grown in a 4 mL rich medium containing 2 mM IPTG for 4 h, centrifuged at 4,000 rpm for 10 min, and removed the supernatant, added in 100 μL LB. Then, positive clones were selected from LB agar medium containing 50 μg/mL kanamycin incubated overnight at 37°C. Screening primers outside the homology region were used to confirm the insertion of the kanamycin cassette (20). Then the following steps were made to make the kanamycin cassette lost in the AB43 Δ*csy1*::kan mutant strains. First, pAT03 (pMMB67EH with flippase recombinase) was electrotransformed into AB43 Δ*csy1*::kan mutant strains. Then, the bacteria were resuscitated and cultured using LB with 2 mM IPTG for 1 h. Positive clones were then selected on LB agar plates containing carbenicillin (75 μg/mL) incubated overnight at 37°C. The *csy1* gene knock-out mutation was confirmed by PCR and DNA sequencing (Tsingke Biotechnology Co., China).

### Complementation of Δ*csy1* Mutant Strain

The pMMB67EH vector was utilized to generate a *csy1* gene complementation vector. An 1,110 bp fragment containing an open reading frame of *csy1 gene* from the genome was amplified and ligated to the pMMB67EH vector, then the recombinant plasmid was electrotransformed into the AB43Δ*csy1*. Complementation vector-transformed Δ*csy1* mutants were selected on LB agar plates containing 50 μg/mL kanamycin and 10 μg/mL tetracycline. Positive clones were verified by colony PCR using primers and DNA sequencing (Tsingke Biotechnology Co, China). The expression of the *csy1* gene in AB43, complemented mutant, and Δ*csy1* mutant strains was determined by qRT-PCR as described above.

### Antimicrobial Susceptibility Testing of Mutant Strains and Resistance Development Studies

For *in vitro* resistance development of AB43, AB43Δ*csy1/pcsy1*, and AB43Δ*csy1*, antibiotics sensitive to all three strains were selected, and then resistance development experiments were conducted. Strains were exposed separately to polymyxin B and rifampin for step-wise selection. Strains at the exponential phase were diluted 1: 1,000 into fresh MHB medium supplement with 0.5 × MIC or 0.25 × MIC of polymyxin B and rifampin. After being cultured at 37°C for 24 h at 200 rpm, the MIC of each drug was determined by broth microdilution as mentioned before. The process was repeated for 10 generations. Moreover, the ratio of the MIC obtained from every generation to the MIC at the first generation (first contact) was determined. The data are expressed as the relative increase of MIC per generation (21).

### Transcriptomic Analysis

The RNA-Seq analysis of AB43 and AB43Δ*csy1* was performed as proposed by Kesavan et al. (22). Total RNAs of AB43 and AB43Δ*csy1* were extracted from cell cultures at the log phase using an RNA extraction kit (TIANGEN, Beijing, PR China) and quantified using a Nanodrop spectrophotometer (Thermo Scientific, Waltham, MA, USA) by the ratio of absorbance (260 nm/280 nm). cDNA synthesis, library generation, and data analysis were performed by Shanghai Sheng gong Bioengineering Company, and transcriptome sequencing was conducted using Illumina HiSeqTM.

## Results

### Spacer Identification and CRISPR Analysis of *A. baumannii*

CRISPR-Cas Finder was used to determine the number and sequence of spacers in the CRISPR-Cas repeat array of ATCC19606 and AB43. The results are illustrated in Figure 1. The results demonstrate that ATCC19606 carries five CRISPRs (Crispr_1, Crispr_2, Crispr_3, Crispr_4, and Crispr_5), where the number of spacers in the CRISPR locus of Crispr_1, Crispr_2, Crispr_3, Crispr_4, and Crispr_5 is 1, 18, 45, 1, and 1, respectively. The Type I-Fa Cas cluster consists of Cas1, Cas6, Csy3, Csy2, and Cas3-Cas2 (Cas3). Strain AB43 carries one confirmed CRISPR, which contains 105 spacers, and the Type I-Fb Cas cluster contains Cas1, Cas3-Cas2 (Cas3), Csy1, Csy2, Csy3, and Csy4.

### Antimicrobial Susceptibility of Selected Strains

The status of the CRISPR-Cas system in selected *A. baumannii* strains is demonstrated in Table 2. To investigate the relationship between *csy1* gene and drug resistance, the relationship between the CRISPR-Cas carrier rate and drug resistance was first investigated. The strains with a complete CRISPR-Cas system (ATCC19606 and AB43) were sensitive to 12 antibiotics tested. However, strains with an incomplete set of CRISPR-Cas-related genes (A133 and F7) or without the CRISPR-Cas system (E5) were resistant to most of the antibiotics tested. The results are shown in Table 3. Thus, the CRISPR-Cas system was speculated to be related to the drug resistance of *A. baumannii*.

**Table 2:** Strains with different carrying situations of CRISPR-Cas system genes.

**Strain**	**ST**	***cas1***	***cas3***	***cas6***	***csy1***	***csy2***	***csy3***	***csy4***
ATCC19606 (I-Fa)	ST931	+	+	+	–	+	+	–
AB43 (I-Fb)	ST705	+	+	–	+	+	+	+
AB133 (I-Fa)	ST1145	+	–	–	–	+	–	–
ABF7 (I-Fb)	ST195	+	–	+	+	+	+	+
E5	ST195	–	–	–	–	–	–	–

*+, contain; –, lack*.

**Table 3:** Minimum inhibitory concentrations (MICs) of strains with different types of CRISPR-Cas system.

**Drugs (μg/ml)**	**ATCC19606**	**AB43**	**A133**	**F7**	**E5**
Doxycycline	0.125(S)	0.125(S)	64(R)	32(R)	32(R)
Minocycline	0.0624(S)	0.015625(S)	32(R)	8(I)	2(S)
Tigecycline	2(S)	0.5(S)	64(R)	8(I)	8(I)
Ceftriaxone	8(S)	2(S)	≥8192(R)	256(R)	1024(R)
Imipenem	8(S)	8(S)	≥512(R)	512(R)	≥512(R)
Gentamicin	8(S)	0.5(S)	1024(R)	≥8192(R)	≥8192(R)
Kanamycin	8(S)	4(S)	≥8192(R)	≥8192(R)	≥8192(R)
Ciprofloxacin	0.25(S)	1(S)	256(R)	16(R)	32(R)
Polymyxin B	0.25(S)	0.125(S)	2(S)	2(S)	2(S)
Colistin	0.125(S)	0.125(S)	0.25(S)	0.125(S)	0.125(S)
Erythromycin	8(S)	1(S)	256(R)	1(S)	4(S)
Rifampin	1(S)	0.5(S)	64(R)	2(S)	2(S)

*R represents resistance; S represents sensitivity*.

### CRISPR-Cas Gene Expression in ATCC19606 and AB43 Exposed to Antibiotics

**Figure 2:**
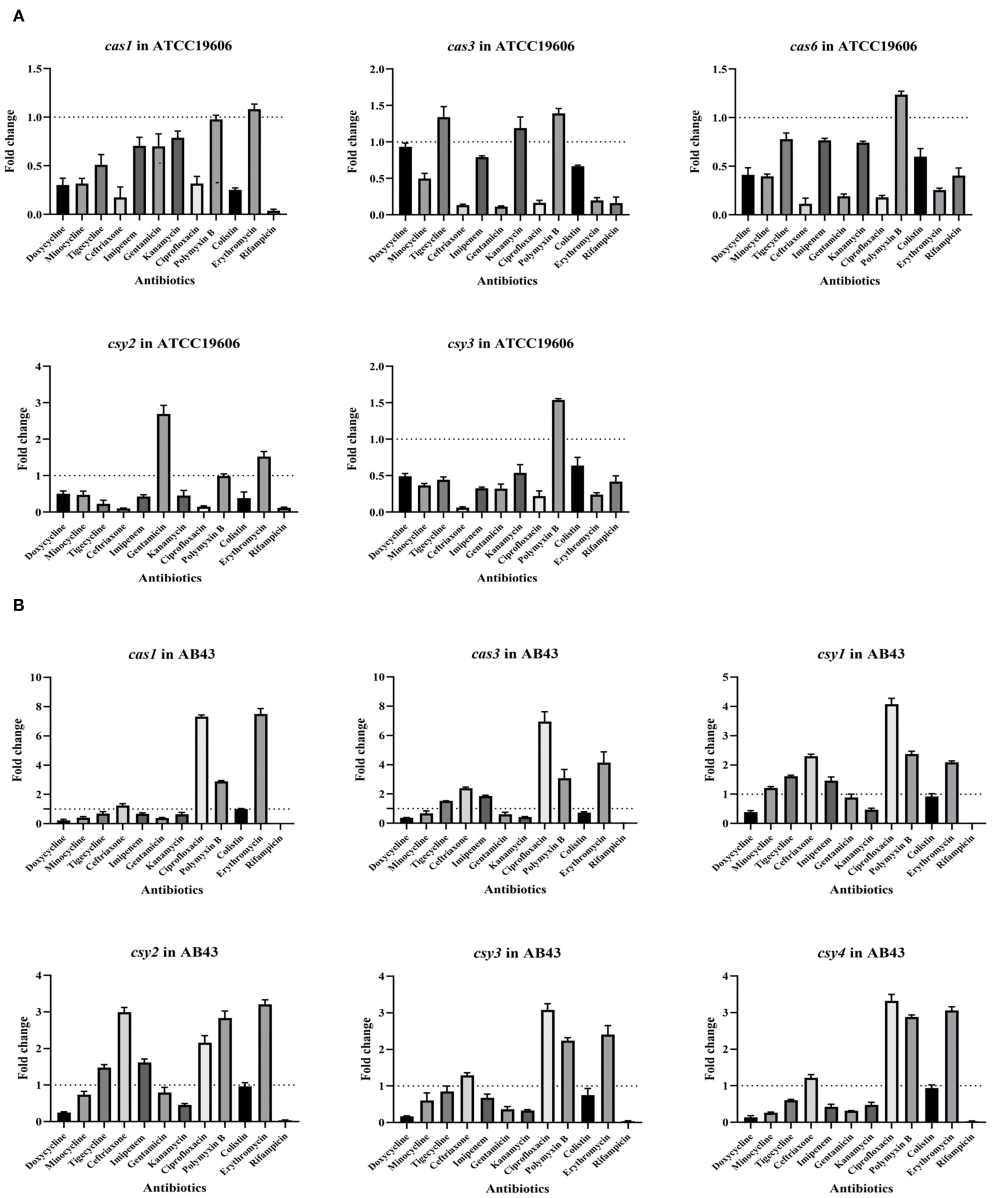
CRISPR-cas gene expression in strains after exposure to antibiotics. **(A)** CRISPR-related genes (*cas1, cas3, cas6, csy2*, and *csy3*) expression in ATCC19606 against antimicrobials; **(B)** CRISPR-related genes (*cas1, cas3, csy1, csy2, csy3*, and *csy4*) expression against antimicrobials in AB43.

qRT-PCR analysis of CRISPR-Cas-related *cas* and *csy* genes was performed to further study the relationship between the CRISPR-Cas system and antimicrobial resistance. The expression of the CRISPR-Cas-related genes *cas* and *csy* changed under antibiotic pressure (Figures 2A,B). Downregulation of *cas1, cas3, cas6, csy2, csy3*, and *csy4* was detected in ATCC19606 and AB43 when treated with most antibiotics. However, *csy1* gene in AB43 was upregulated when treated with most antibiotics, and only downregulated when treated with doxycycline and kanamycin.

### The Role of *csy1* Gene in Antimicrobial Susceptibility

**Figure 3:**
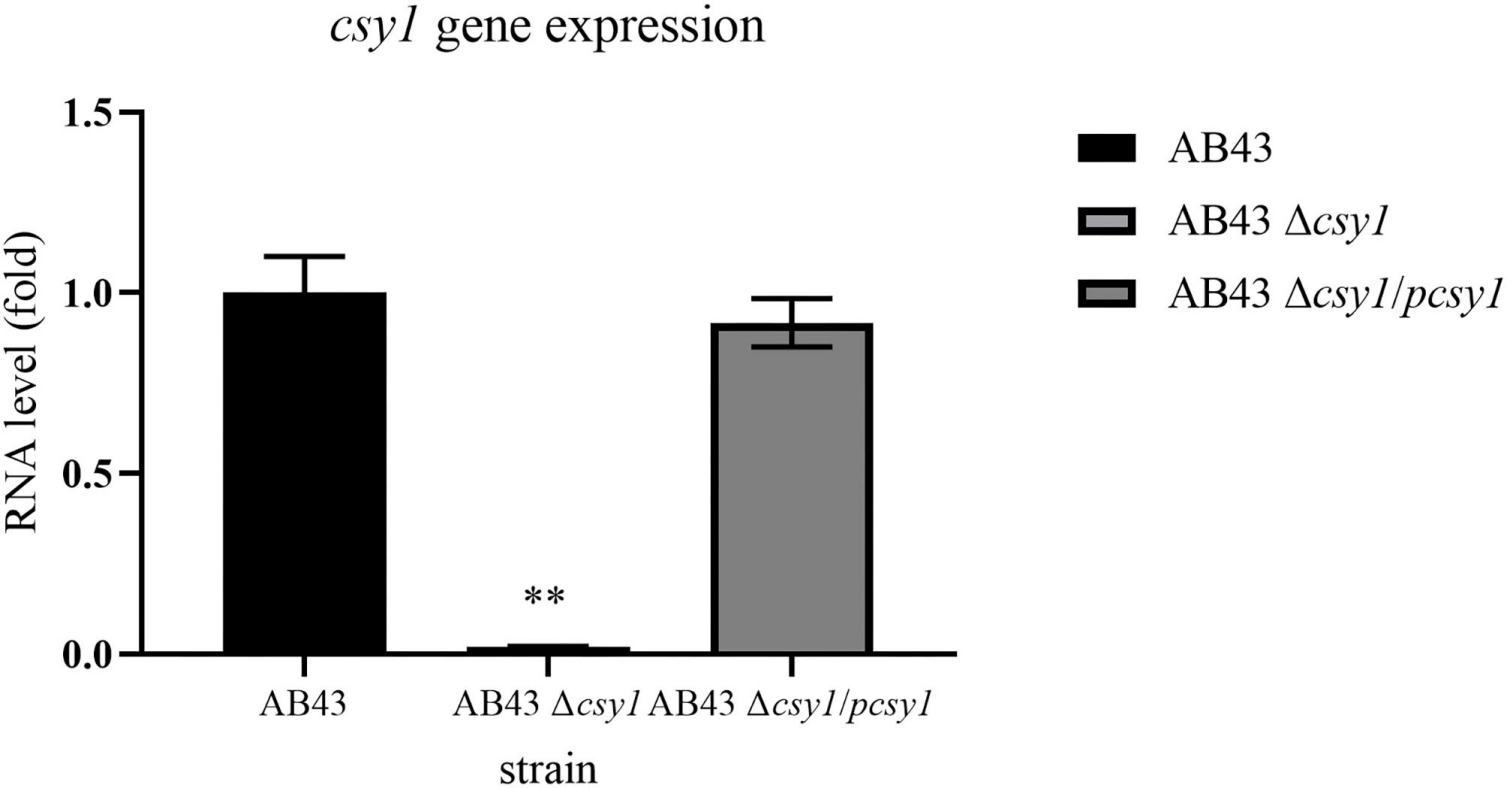
The RNA level of *csy1* gene in wild type (AB43), *csy1 gene* deletion (AB43Δ*csy1*), *csy1gene* complementation (AB43Δ*csy1/pcsy1*) strains. The RNA level in AB43 was set as 1, and those in strains AB43Δ*csy1* and AB43Δ*csy1/pcsy1* were calculated accordingly. Data are presented as means ± SEM from three independent experiments. **p < 0.01.

The above studies demonstrated that an integrated CRISPR-Cas system might inhibit the drug resistance of *A. baumannii*. Further, when *A. baumannii* was exposed to tested antibiotics, only *csy1* gene was upregulated. However, little is known regarding the role of *csy1* gene in the drug resistance of *A. baumannii*. A *csy1* gene knock-out strain was constructed. The expressions of *csy1* gene RNA in the wild type (AB43), *csy1* gene deletion (AB43Δ*csy1*), and *csy1* gene complementation (AB43Δ*csy1/pcsy1*) strains were determined by qRT-PCR. The expression of *csy1* gene RNA was significantly decreased by deletion in the *csy1* gene deletion mutant, and restored to wild-type levels in the *csy1* gene complementation strain (Figure 3). The antimicrobial susceptibility of AB43, the complemented mutant, and Δ*csy1* mutant strains to 12 drugs was evaluated. The results are shown in Table 4. A 16- to 512-fold (doxycycline, tigecycline, ceftriaxone, imipenem, ciprofloxacin, erythromycin) increase in susceptibility was observed in the Δ*csy1* mutant strain compared with its wild (AB43) strain. Moreover, a >1,024-fold increase of MIC (minocycline, gentamicin, and kanamycin) was observed in the Δ*csy1* mutant strain compared with AB43. The MICs of polymyxin B, colistin, and rifampin were similar in the three strains.

**Table 4:** Minimum inhibitory concentrations (MICs) of AB43, complemented mutant, and Δ*csy1* mutant strains to different antibiotics.

**Drugs (μg/ml)**	**AB43**	**AB43Δ***csy1/***p***csy1*****	**AB43Δ** ***csy1***
Doxycycline	0.125(S)	0.015625(S)	16(R)
Minocycline	0.015625(S)	0.015625(S)	16(R)
Tigecycline	0.5(S)	8(S)	128(R)
Ceftriaxone	2(S)	4(S)	256(R)
Imipenem	8(S)	8(S)	≥4096(R)
Gentamicin	0.5(S)	1(S)	≥4096(R)
Kanamycin	4(S)	4(S)	≥4096(R)
Ciprofloxacin	1(S)	0.0625(S)	16(R)
Erythromycin	1(S)	0.5(S)	64(R)
Colistin	0.125(S)	0.0625(S)	0.25(S)
Polymyxin B	0.125(S)	0.125(S)	0.125(S)
Rifampin	0.5(S)	0.5(S)	1(S)

*R represents resistance; S represents sensitivity*.

### The Role of csy1 Gene in Resistance Development

**Figure 4:**
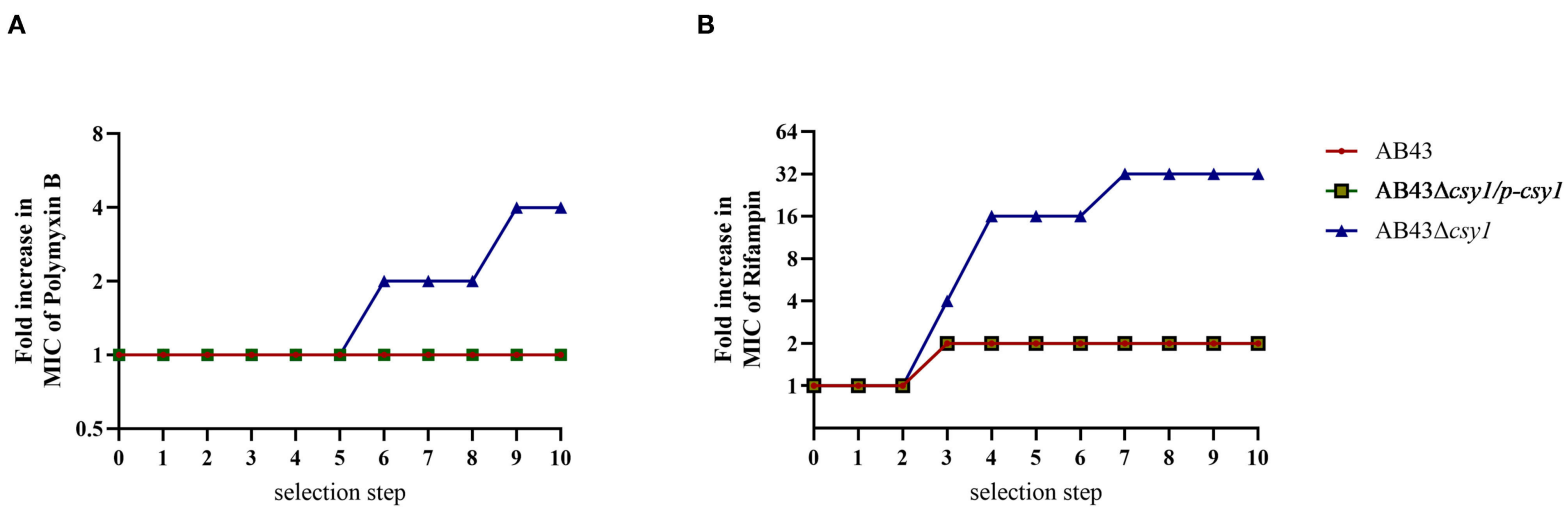
Polymyxin B and rifampicin resistance gains during continuous passaging. **(A)** Polymyxin B; **(B)** Rifampicin.

Based on the results that *csy1* gene can regulate the drug resistance of *A. baumannii*, a drug resistance development assay was done to further elucidate its role in antibiotic resistance. No significant differences were found between AB43, AB43Δ*csy1*/p*csy1*, and AB43Δ*csy1* in resistance to polymyxin B and rifampin (Table 4); thus, both were chosen for the resistance induction experiment. There was no significant difference among AB43, AB43Δ*csy1/pcsy1*, and AB43Δ*csy1* in resistance to polymyxin B until the sixth step of selection (2 μg/mL). At 2 μg/mL polymyxin B, only AB43Δ*csy1* exhibited growth (Figure 4). The drug resistance of rifampicin showed a difference at the third selection step and a 16-fold increase at the eighth selection step (Figure 4). The results suggested that the *csy1* gene mutant strain drug resistance developed significantly faster than that of the wild strain.

### Transcriptomic Analysis of the *csy1* Gene Knock-Out Mutant

**Figure 5:**
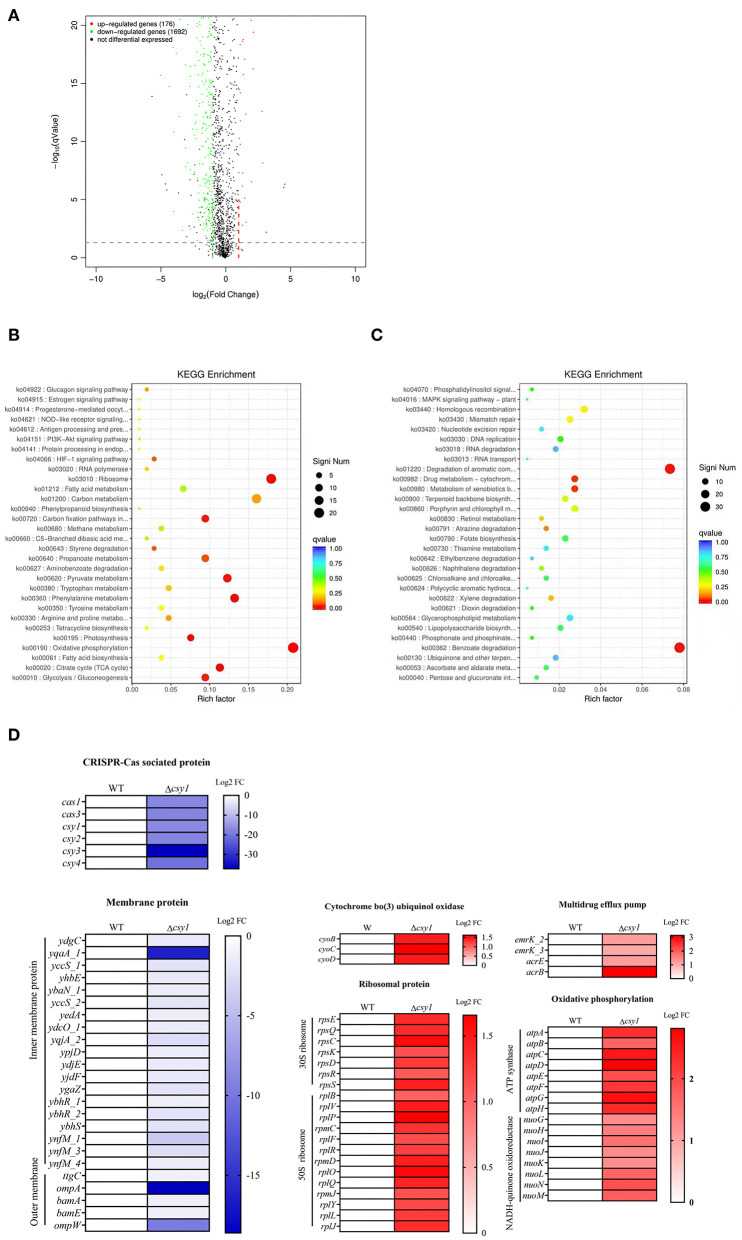
Transcriptome analysis of AB43 and AB43Δ*csy1*. **(A)** Differential gene volcano map; **(B)** Kyoto Encyclopedia of Genes and Genomes (KEGG) enrichment analysis of up-regulation genes; **(C)** KEGG enrichment analysis of down-regulation genes; the color of the dot represents the size of the Q-value, and the size of the dot represents the number of differential genes; **(D)** Selected differential expression genes involved in CRISPR-Cas related protein, ribosome protein, oxidative phosphorylation related protein, a membrane-associated related protein, and multidrug efflux pump protein.

Transcriptome analyses of AB43 and AB43Δ*csy1* were performed, which demonstrated that genes encoding efflux protein, the 50S/30S ribosomal proteins, proteins involved in energy metabolism, and cytochrome C were upregulated in AB43Δ*csy1* (Figure 5). The upregulation of the gene encoding the efflux pump was reported to be associated with increased antimicrobial resistance (23). Additionally, the increased synthesis of ATP could promote drug efflux. Several downregulated genes in the mutant strain were also detected. After the *csy1* gene was knocked out, the expression of CRISPR-Cas-related genes was downregulated. The related proteins of the CRISPR-Cas system were speculated to influence each other as a whole. The decreased expression of membrane-related proteins indicated that the CRISPR-Cas system might regulate cell membrane formation.

## Discussion

The primary role of the CRISPR-Cas system is bacterial defense against phages, plasmids, and other foreign DNA. Some recent studies showed that the CRISPR-Cas system might play a role in antibiotic resistance of bacteria (24). The effect of the CRISPR-Cas system on antibiotic resistance varies in different bacteria. The CRISPR-Cas system promotes resistance in *C. jejuni* and inhibits resistance in *E. coli* and *E. fecium* (8, 9, 25). However, the relationship of the CRISPR-Cas system with antibiotic resistance in *A. baumannii* is not fully elucidated. By investigating the carrying situation of the CRISPR-Cas system and the drug resistance level of selected strains, we assessed whether the CRISPR-Cas system plays a role in *A. baumannii* antibiotic resistance. The results showed that the complete CRISPR-Cas system might inhibit bacterial drug resistance. qRT-PCR analysis was conducted to measure the expression levels of Type I-Fa and I-Fb CRISPR-Cas system-related genes after antibiotic treatment. The results demonstrated that the expressions of *cas* and *csy* genes were modified.

With the excessive use of antibiotics, the bacterial resistance rate has continued to increase. The infection rate of MDR pathogens is increasing. Thus, the development of different strategies to combat antimicrobial resistance is critical (4). Moreover, research on the relationship between CRISPR-Cas and bacterial resistance is primarily focused on the *cas3* and *cas9* genes (26). The *cas3* gene in *Streptococcus* mutants can regulate biofilm formation and the ability to resist fluoride (27). Sampson et al. proved that the CRISPR-Cas endonuclease gene *cas9* together with tracrRNA and ScaRNA is essential for enhancing the stability of the bacterial envelope and promoting antibiotic resistance (10). However, the relationship between the *csy1* gene and drug resistance in bacteria has not been previously reported. To the best of our knowledge, this is the first study regarding the relationship between *csy1* gene and drug resistance in *A. baumannii*. Preliminary studies have demonstrated that *csy1* gene can regulate the drug resistance of *A. baumannii* and maintain its sensitivity to antibiotics. After knocking out the *csy1* gene, the bacterial resistance level increased. Simultaneously, the drug resistance experiment showed that *csy1* gene could inhibit the drug resistance development of bacteria. The complete CRISPR-Cas system may inhibit its drug resistance. The results are similar to studies on *Enterococcus fecalis*, which found that the lack of CRISPR genes is related to species, multidrug resistance, and major drug resistance-related genes (28).

The study by Aydin et al. suggested that the CRISPR-Cas system may interfere with the acquisition of resistant plasmids, thereby maintaining the sensitivity of these strains (9). *Klebsiella pneumoniae* strains detected that the spacers in the CRISPR array matched the genome of the plasmid or phage, some containing resistance genes (29). In *Francis bacteria*, CRISPR-Cas can enhance the integrity of its envelope, leading to resistance to several membrane stressors, including antibiotics, and increasing antibiotic resistance (10). Transcriptome analysis was conducted to assess the mechanism of *csy1* gene inhibiting the drug resistance of *A. baumannii*. The *csy1* gene mutant strain has a higher expression of drug efflux pump, ATP synthesis-related, and ribosomal genes, as well as a lower expression of CRISPR-Cas-related and membrane-related genes.

Thus, with the steady increase in MDR bacterial infections, the innovation of novel therapies to combat MDR bacteria is critical. Existing studies have demonstrated that the CRISPR-Cas system delivered by phages can sequentially eliminate *Staphylococcus aureus* (30). CRISPR-encoded presentation plasmids or CRISPR-Cas antibacterial drugs can reduce the occurrence of antibiotic resistance in *Enterococci* (31). The delivery of CRISPR-Cas9 effectively removes antibiotic resistance *in vitro* by targeting plasmid-borne resistance genes (32). Our work main revealed that the *csy1* gene participates in the regulation of drug resistance in *A. baumannii*. The present study has several limitations. It is unknown how *csy1 gene*, regulates drug resistance in *A. baumannii*. Moreover, we only performed *csy1* gene knock-out experiments in AB43 (I-Fb CRISPR-Cas), and more strains are needed to generalize our results. Thus, future studies should focus on assessing the mechanism by which CRISPR-Cas regulates drug resistance. Moreover, deploying the Type I CRISPR-Cas system as an antimicrobial to treat drug-resistant *A. baumannii* infection is an attractive strategy compared with conventional antibiotic therapy. It is also necessary to explore the effect of other CRISPR-Cas components in modulating aspects of bacterial physiology such as virulence and drug resistance.

## Conclusions

In conclusion, we studied the relationship of the CRISPR-Cas system with antibiotic resistance in *A. baumannii*. Our results revealed that the expression of CRISPR-Cas related genes was changed under antibiotic pressure and that the presence of the *csy1* gene has an inhibitory effect on the drug resistance of *A. baumannii*. Deletion of the *csy1* gene in *A. baumannii* strain AB43 made it resistant to most of the antibiotics tested. In addition, this study extends our understanding of resistance regulation of *A. baumannii* and provides a new direction for studying the functions of CRISPR-Cas systems in drug resistance. Future work can be focused on their functions that extend beyond the general disruption of invading foreign DNA and may clarify how CRISPR-Cas systems contribute to bacterial resistance and virulence. This knowledge will be useful in the exploration of new antibacterial strategies.

## Data Availability Statement

The datasets presented in this study can be found in online repositories. The names of the repository/repositories and accession number(s) can be found at: NCBI SRA; SRR17253291 and SRR17253292.

## Author Contributions

GL and TG contributed to the design of the study. TG, XS, ML, and YW contributed to the acquisition of the data. GL, TG, and XS contributed to the analysis of the data. All authors contributed to data interpretation, drafting the manuscript, critically revising the manuscript for important intellectual content, and approved the final version of the manuscript.

## Funding

This work was supported by the National Natural Science Foundation of China (82073611 and 82002186), the Natural Science Foundation of the Higher Education Institutions of Jiangsu Province (Grant Number: 19KJB310002).

## Conflict of Interest

The authors declare that the research was conducted in the absence of any commercial or financial relationships that could be construed as a potential conflict of interest.

## Publisher's Note

All claims expressed in this article are solely those of the authors and do not necessarily represent those of their affiliated organizations, or those of the publisher, the editors and the reviewers. Any product that may be evaluated in this article, or claim that may be made by its manufacturer, is not guaranteed or endorsed by the publisher.
