# Supplementary material for: The Involvement of the csy1 Gene in the Antimicrobial Resistance of Acinetobacter baumannii
Source: Front Med (Lausanne). 2022 Jan 26;9:797104. doi: 10.3389/fmed.2022.797104 (PMC8825777; doi:10.3389/fmed.2022.797104)
Supplement: Supplementary file 1 [file Table_1.DOCX]

**Table S1.** The list of primers used in the CRISPR-Cas genes detection. All primer sequences are in 5’-3’ orientation.

| Genes | Sequences (5′-3′) | Length (bp) |
| --- | --- | --- |
| IFa-cas1-F  IFa-cas1-R | GATCGAATTGTTTACTTGACGGAG TATCTTGGTCTTGGCATGTTTC | 522 |
| IFa-cas3-F  IFa-cas3-R | AGTAGCCACCCGACAAAC  CATTTGCATACCCGACTC | 3237 |
| IFa-csy2-F  IFa-csy2-R | CGATGAGTCGTTATGTCGTTATTCCTCG  AAAAATGTCGTCTTGCGGCCAG | 857 |
| IFa-csy3-F  IFa-csy3-R | CTTTACAACGCGGTACAGTGGTGA  TTATTCACCCTCGGAGTACACGCC | 1028 |
| IFa-cas6-F  IFa-cas6-R | GGATATCCGTGTTCTTGAGTCATCAG  GACGGGTAGGGCAAAAGGAC | 583 |
| IFb-cas1-F  IFb-cas1-R | AAGCTGCGATGCGAATGTTATGTA  CTATGGCTAATTCCTAGTACCCAA | 807 |
| IFb-cas3-F  IFb-cas3-R | GAACAGGTGAAGTTGCAAGCA  ATGGCACTTTCATTTTCGGC | 3531 |
| IFb-csy1-F  IFb-csy1-R | ACAACTTACCTTTTCCGAGCA  AACTCGCCTGTTCCTAACGT | 1256 |
| IFb-csy2-F  IFb-csy2-R | TCAGGATTATTACGCCAGCCT  CGATTTCCCCATGACGTACCA | 1244 |
| IFb-csy3-F  IFb-csy3-R | GGAGTTTGTGATGCCATACCG  TCGGCAAAAGCAATATGAAGC | 1250 |
| IFb-csy4-F  IFb-csy4-R | GGCTGTTTTAGTACGTGGTGG  TCGATTGCAACAACTTACTCGT | 843 |

**Table S2** Primers used for qRT-PCR. All primer sequences are in 5’-3’ orientation.

| Strain | Genes | Sequence (5' to 3') | Length (bp) |
| --- | --- | --- | --- |
|  | 16s rRNA-F | GTCGTCCCCGCCTTCCT | 93 |
|  | 16s rRNA-R | AGCATTTCGGATGGGAACTTTA |  |
| ATCC19606 | q*cas1*-F | GGCAGCCGCATCTGTTAATG | 173 |
|  | q*cas1*-R | CAAAGCGTGCCTGCGTATTT |  |
|  | q*cas3*-F | ACCAATGACAATGGACGGCT | 156 |
|  | q*cas3*-R | GCACCGTCATGGGCAAATTT |  |
|  | q*cas6*-F | CCATAGCTATCTGGCAGCCC | 119 |
|  | q*cas6*-R | AAAACGGCCAAAGGAATGCC |  |
|  | q*csy2*-F | CCAATAAACACGGCACCACG | 165 |
|  | q*csy2*-R | GTGGTGTGCCTTTGTTTGCA |  |
|  | q*csy3*-F | CACCACCCATTCCGAAGTCA | 243 |
|  | q*csy3*-R | ATCGTGAATGGTCGCTGGTT |  |
| AB43 | q*cas1*-F | AGTTTTGAACGGAACCCTGAGCAG | 91 |
|  | q*cas1*-R | GTGTCGTCGCACTTAGGCCATAG |  |
|  | q*cas3*-F | GCCAAGACTTGATTGCGATTGCC | 137 |
|  | q*cas3*-R | TAACGACCAACCGTGCTGATAAGC |  |
|  | q*csy1*-F | GGCCGAAAATGAAAGTGCCATAGC | 83 |
|  | q*csy1*-R | CTGCGTCAGGAAGCCACTCATTC |  |
|  | q*csy2*-F | CAGGGGCGTGATGCTTTAGATGC | 117 |
|  | q*csy2*-R | AAGCCAGCCGTGCGTTTGAC |  |
|  | q*csy3*-F | AGGATGTTGCTGCAATGCACTCTC | 117 |
|  | q*csy3*-R | CCGCACCGTATGGCTCAATCGG |  |
|  | q*csy4*-F | GTGTTTGCCAGCTCGGAAAATGAC | 117 |
|  | q*csy4*-R | TGTAATCTTGGCTCGAGGCACTTC |  |
